# Factor H: A Complement Regulator in Health and Disease, and a Mediator of Cellular Interactions

**DOI:** 10.3390/biom2010046

**Published:** 2012-02-07

**Authors:** Anne Kopp, Mario Hebecker, Eliška Svobodová, Mihály Józsi

**Affiliations:** Junior Research Group Cellular Immunobiology, Leibniz Institute for Natural Product Research and Infection Biology, Jena 07745, Germany; Email: anne.braunschweig@hki-jena.de (A.K.); mario.hebecker@hki-jena.de (M.H.); eliska.svobodova@hki-jena.de (E.S.)

**Keywords:** complement, factor H, pentraxin, inflammation, apoptotic cell, cell adhesion, extracellular matrix

## Abstract

Complement is an essential part of innate immunity as it participates in host defense against infections, disposal of cellular debris and apoptotic cells, inflammatory processes and modulation of adaptive immune responses. Several soluble and membrane-bound regulators protect the host from the potentially deleterious effects of uncontrolled and misdirected complement activation. Factor H is a major soluble regulator of the alternative complement pathway, but it can also bind to host cells and tissues, protecting them from complement attack. Interactions of factor H with various endogenous ligands, such as pentraxins, extracellular matrix proteins and DNA are important in limiting local complement-mediated inflammation. Impaired regulatory as well as ligand and cell recognition functions of factor H, caused by mutations or autoantibodies, are associated with the kidney diseases: atypical hemolytic uremic syndrome and dense deposit disease and the eye disorder: age-related macular degeneration. In addition, factor H binds to receptors on host cells and is involved in adhesion, phagocytosis and modulation of cell activation. In this review we discuss current concepts on the physiological and pathophysiological roles of factor H in light of new data and recent developments in our understanding of the versatile roles of factor H as an inhibitor of complement activation and inflammation, as well as a mediator of cellular interactions. A detailed knowledge of the functions of factor H in health and disease is expected to unravel novel therapeutic intervention possibilities and to facilitate the development or improvement of therapies.

## 1. Introduction: The Complement System

Innate immunity is a first-line defense system, essential for the protection of the host against invading pathogens, acting immediately after infection and without previous antigen contact [1]. The innate immune system comprises several cellular and humoral components and utilizes germ-line encoded receptors for the recognition of microorganisms. A major humoral component of innate immunity is the complement system, which was established early in evolution and is present in invertebrates lacking an adaptive immune system. Originally identified as a serum component that ‘complements’ the antibody response towards pathogens, it is now known as a system of more than forty proteins. These proteins form a complex network of various recognition, effector, regulatory and receptor molecules that act in a finely tuned fashion, allowing complement to safely exert its functions [2]. It is well acknowledged that the functions of the complement system go far beyond the elimination of invading microbes. In addition to protecting the host from infections by destroying pathogens and promoting their elimination, complement has important roles in maintaining the integrity of the body by discriminating between healthy and injured tissue, in participating in the disposal of immune complexes, apoptotic/necrotic cells and cellular debris, as well as in inflammatory processes, angiogenesis and tissue regeneration. Moreover, complement is involved in the induction and regulation of both innate and adaptive cellular immune responses [3,4].

**Figure 1:**
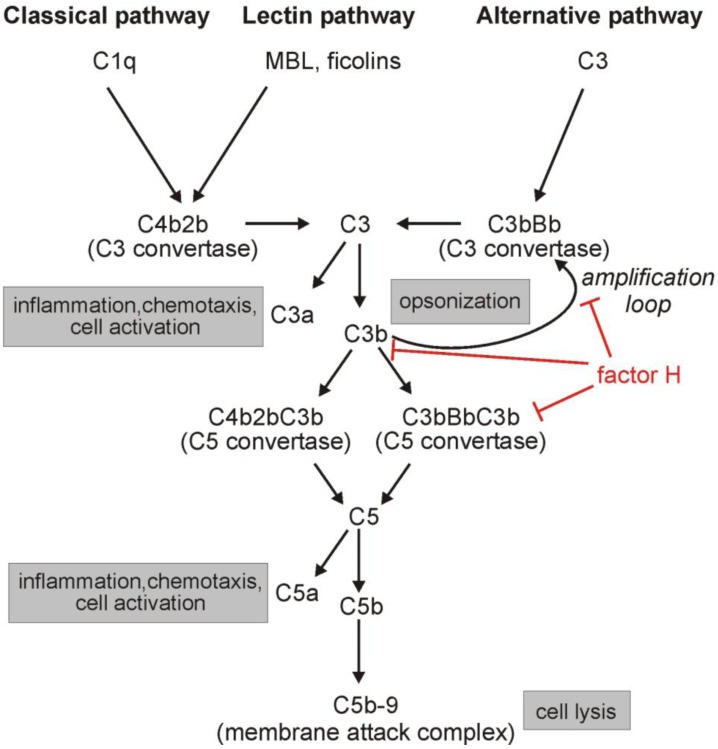
Complement activation pathways and the regulatory role of factor H.Complement activation is initiated by the recognition molecules of the classical (C1q) and lectin (MBL, ficolins) pathways or by the hydrolysis of the C3 thioester bond (alternative pathway). All three activation cascades lead to the assembly of C3 convertase enzymes that cleave the central C3 molecule into C3a and C3b. C3b deposits to nearby surfaces and, if not inactivated, it forms additional C3 convertases (amplification loop). The binding of C3b to an existing C3 convertase results in a C5 convertase that cleaves C5 into C5a, a potent anaphylatoxin, and C5b. C5b binds to surfaces and by binding C6, C7, C8 and several C9 molecules a terminal membrane attack complex is formed that allows target cell lysis. The system is controlled by several fluid phase and membrane-bound regulators that act at various steps of the activation cascade. Factor H is the major fluid-phase regulator of the alternative pathway, as it prevents the formation of the C3 and C5 convertases, facilitates the disassembly of already formed convertases and acts as a cofactor for the inactivation (enzymatic cleavage) of C3b.

Complement can be activated via three major pathways that merge at the central component C3 (Figure 1). The classical pathway is initiated by C1q binding to immune complexes, pentraxins or other targets such as apoptotic cells, the lectin pathway by binding of mannan-binding lectin (MBL) to repetitive carbohydrate residues, or by binding of ficolins to carbohydrate or acetylated groups on target surfaces, and the alternative pathway is spontaneously autoactivated by the hydrolysis of the internal thioester group of C3. Activation of the three pathways leads to the generation of the classical/lectin pathway C3 convertase (C4b2b) and the alternative pathway C3 convertase (C3bBb). These enzymes cleave C3, resulting in the two main fragments C3a, a potent inflammatory mediator, and C3b, an opsonin that deposits on the surface of target cells and particles and promotes phagocytosis. C3b can also form additional C3 convertases and in this way amplify complement activation. Furthermore, when C3b binds to an already existing C3 convertase, the new complex is termed a C5 convertase as it gains the ability to cleave C5 and thus to activate the terminal complement pathway. This potentially leads to inflammation (via C5a generation) and target cell lysis through the formation of membrane pores by the C5b-9 membrane attack complex. Whereas these activation processes are strongly favored on microbial surfaces, they are potentially destructive to host cells. To prevent host tissue damage, the activation of the complement system is strictly regulated by membrane-bound and plasma regulatory molecules. Since the alternative pathway is constitutively activated at a background level and is also activated secondary to classical/lectin pathway activation (through the ‘amplification loop’), its regulation is particularly important. Factor H is a major plasma regulator acting in the alternative complement pathway at the level of the central C3b component, facilitating C3b inactivation and the dissociation of the C3/C5 convertases and thus blocking further activation of the complement cascade (Figure 1).

Disturbances in this complex system caused by complement gene mutations, autoantibodies or exogenous triggers may tip the balance between complement activation and inhibition, resulting in an attack on self tissue [4,5]. Complement deficiencies and malfunctions in the complement system are associated with various infectious, inflammatory and (auto)immune diseases. Factor H gene mutations and polymorphisms, as well as anti-factor H autoantibodies, are associated with several diseases that are characterized by complement dysregulation, e.g., the eye disorder age-related macular degeneration (AMD) and the rare kidney diseases atypical hemolytic uremic syndrome (aHUS) and dense deposit disease (DDD) [6,7,8]. Significant progress has been made in recent years in clarifying the roles of factor H and complement in these pathological conditions. Novel ligands and functions of factor H have been identified. Beside its role as a complement regulator, factor H has been shown to mediate cellular interactions by binding to receptors on various cells. A detailed description of the physiological and pathophysiological roles of factor H and its intricate interactions with other plasma and cell membrane molecules are necessary for our understanding of the underlying pathomechanisms of inflammatory, autoimmune and infectious diseases where factor H is implicated. This could lead to improved diagnostics and to the development of more effective treatments for affected patients. 

This review summarizes the current concepts of the roles of factor H in health and disease and discusses open questions for future research.

## 2. The Complement Regulator Factor H: Structure and Function

Factor H is the main soluble regulator of the alternative complement pathway [9,10]. The factor H gene (*CFH*) is located on chromosome 1q32 in the regulators of complement activation (RCA) gene cluster, adjacent to the five genes that code for the factor H-related proteins (CFHRs). Factor H is constitutively expressed in the liver and is distributed systemically in body fluids. Reported factor H plasma concentrations vary, depending on the study population, age, genetic and environmental factors, as well as on the method used for quantification [11,12,13,14]. Of note, previous studies overestimated factor H concentration: a result of measuring both factor H and factor H-related proteins. While early reports suggested that the plasma factor H concentration ranges 265–684 µg/mL [11] and 116–562 µg/mL [12], current studies using monoclonal antibodies have established mean factor H concentrations of 233 µg/mL (in young adults), 269 µg/mL (in elderly individuals) [13], and 263 µg/mL [14], in different control populations. Thus, normal factor H concentrations in human plasma correspond to approximately 1–2 µM. In addition, factor H is produced extrahepatically by different cell types such as monocytes [15], fibroblasts [16], endothelial cells [17], keratinocytes [18], platelets [19] and retinal pigment epithelial cells [20]. Locally released factor H in tissues may help to limit complement activation and maintain an anti-inflammatory environment.

**Figure 2:**
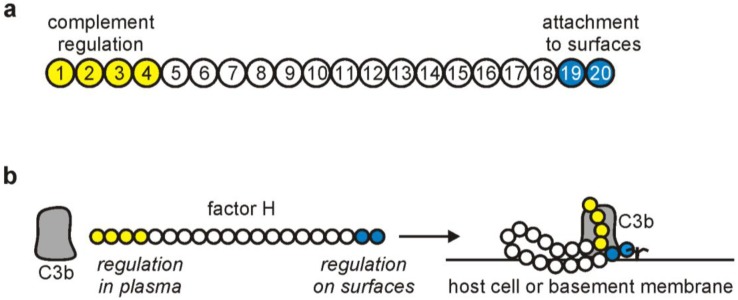
The schematic structure of factor H. **(a)** Factor H is composed of 20 short consensus repeat (SCR) domains. Two major functional regions are located at the N- and C-termini of the molecule. SCRs 1-4 mediate the complement regulatory activities of factor H; **(b)** The SCRs 19-20 allow the attachment of factor H to host cells so that it can also inhibit complement activation directly at the cell surface.

Factor H is a single-chain, 150-kDa plasma glycoprotein composed of 20 domains. These are termed short consensus repeats (SCRs) or complement control protein modules (CCPs). Each of these autonomously folding globular domains is composed of approximately 60 amino acids and is stabilized by two internal disulfide-bonds [21]. Factor H regulates complement activation by (i) inhibiting the assembly of the alternative pathway C3 and C5 convertase enzymes via competition with factor B for C3b binding; (ii) facilitating the disassembly of the convertases by displacing bound factor Bb (‘decay accelerating activity’); and (iii) acting as a cofactor for the serine protease factor I in the cleavage and inactivation of C3b (‘cofactor activity’) [22,23,24]. These regulatory activities are mediated by the four N-terminal domains SCRs 1–4 [25,26], while the C-terminal domains SCRs 19-20 are responsible for target recognition (Figure 2) [27,28]. One of the important targets for factor H binding in the vicinity of C3b on host cells are polyanionic surface molecules, such as glycosaminoglycans and sialic acid, which increase the affinity of factor H for C3b [29,30]. Thus, in addition to its regulatory activities in the fluid phase, factor H is also able to control complement activation on self-surfaces (Figure 2) [31,32,33]. In contrast, host-like polyanionic molecules are normally not present on the surface of pathogens, rendering them susceptible to complement attack. 

In addition to C3b and polyanionic molecules (such as surface glycosaminoglycans), factor H interacts with further endogenous ligands (Table 1), including the pentraxins C-reactive protein (CRP) and pentraxin 3 (PTX3), the extracellular matrix (ECM) proteins fibromodulin, osteoadherin and chondroadherin, prion protein, adrenomedullin, DNA, annexin-II and histones. These interactions allow factor H to inhibit complement on certain host surfaces (such as the glomerular basement membrane, the extracellular matrix, and late apoptotic cells), that are otherwise not properly protected due to a reduced expression or the lack of membrane-anchored complement regulators (*i.e.*, membrane cofactor protein, decay accelerating factor and CD59). Several of these ligands and structures activate complement via interactions with C1q, MBL or ficolins. The simultaneous binding of both complement activating molecules (*i.e.*, C1q, MBL) and complement inhibiting molecules (*i.e.*, factor H, C4b-binding protein) on such ligands and cells may facilitate their opsonization and safe removal, but at the same time prevents an exaggerated complement activation which could lead to inflammation, cell lysis and subsequent tissue damage [34]. Factor H also binds to nonhost ligands, such as certain surface proteins of microbes, which hijack host factor H in order to protect themselves from complement attack (see section 6.1.), (reviewed e.g., in [35]). Furthermore, factor H can bind to receptors on host cells and mediate functions unrelated to its regulatory activity in the complement system (see section 7).

**Table 1:** Factor H ligands, binding sites and potential relevance of the interactions.

Ligand		Binding sites	Relevance	References
C3 fragments	C3b	SCRs 1-4, 6-8	Complement regulation	[36,37,38]
	C3c	SCRs 12-14		
	C3d	SCRs 19-20		
Polyanionic molecules	Heparin (Glycosaminoglycans, Sialic acid)	SCRs 7, 19-20	Attachment to host cells	[29,38,39]
Pentraxins	C-reactive protein	SCRs 7, 8-11, 19-20	Targeting the activity of factor H	[40,41,42]
	Pentraxin 3	SCRs 7, 19-20		[43]
Apoptotic/necrotic cells	Annexin-II	SCRs 6-8	Promoting safe clearance, protection from autoimmunity	[41,44,45,46]
	DNA	SCRs 6-8, 19-20		
	Histones	SCRs 1-4, 6-8, 8-15		
Extracellular matrix	Fibromodulin	SCRs 6-8	Regulation of inflammation (e.g., in rheumatoid arthritis)	[47,48,49]
	Osteoadherin	?		
	Chondroadherin	?		
Malondialdehyde		SCRs 7, 20	Protection from oxidative stress	[50]
Prion protein		?	Complement dysregulation may promote inflammation in the brain	[51]
Adrenomedullin		SCRs 8-11, 15-20	Modulation of adrenomedullin functions	[52,53]

## 3. Factor H Interactions with Host Ligands

### 3.1. Factor H Interaction with C3b

The central complement protein C3b is the main host ligand of factor H. The C3b-factor H interaction is of particular importance for the pivotal functions of factor H, namely complement regulation and host surface recognition. In factor H, four binding sites were reported for C3b and its fragments, each with a different binding preference, affinity and functional relevance [36,37,38]. These binding sites are located in the SCR domains 1-4, 6-8, 12-14 and 19-20. Solid evidence supports the two major C3b binding sites in SCRs 1-4 and 19-20, whereas evidence for additional binding sites remains inconclusive. 

The N-terminal SCRs 1-4 mediate factor H binding to intact C3b, SCRs 12-14 to the C3c part of C3b (i.e., binds both C3b and the C3c fragment), and SCRs 19-20 to the C3d part of C3b (*i.e.*, binds both C3b and the C3d fragment) [37]. Surface plasmon resonance analyses indicate that the main binding sites are in SCRs 1-4 and SCRs 19-20, with the latter having the higher affinity [38], whereas additional domains may contribute to C3b binding. The crystal structure of the complex of C3b and factor H SCRs 1-4 showed that all four N-terminal domains of factor H are involved in this interaction [54], and that these domains are necessary and sufficient for both cofactor and decay accelerating activities [25,54].

Self-nonself discrimination by factor H is mediated by SCRs 19-20 that bind to surface-bound C3b/C3d and host surface glycosaminoglycans or sialic acids [28,38]. Recent structural data provided insights into this host recognition mechanism. SCR19 contains a main C3d (and C3b) binding site, whereas glycosaminoglycan binding is mediated by SCR20 [55,56]. Thus, the deposited C3b and the surface glycosaminoglycans (the latter lacking on microbes) together allow factor H SCRs 19-20 the recognition of host cells. Furthermore, the data of Kajander *et al.* raises the possibility that in addition to host polyanionic molecules, factor H is recruited by previously deposited and degraded C3b (*i.e.*, C3d) via the SCR20 domain [55].

### 3.2. Factor H Attachment to Host Cells: An Important Role in Self-Nonself Discrimination by Complement

In addition to membrane-bound regulators, host cells require soluble complement inhibitors, particularly factor H, which provide effective protection from unwanted complement-mediated damage, especially under conditions with strong complement activation. This is exemplified by the attachment of factor H to endothelial cells via cell surface glycosaminoglycans and C3b, as discussed above, which is impaired in aHUS and is associated with endothelial damage and acute renal failure [32,33,55,56]. Also, factor H can bind to cell surface polyanionic molecules in the absence of C3b, although this binding is weak and not readily detectable in physiological buffers [32]. Notably, this interaction is distinct from the binding to specific cellular receptors (see section 7).

Host cells, so-called nonactivators of complement, possess cell surface polyanionic molecules that allow for factor H binding [29]. In contrast, complement activators such as microbes or rabbit erythrocytes that lack sialic acids and host-like glycosaminoglycans do not allow significant factor H binding and thus complement activation can proceed unchecked. Heparin is generally used in studies as a model of host glycosaminoglycans, and the major heparin binding sites were located in SCRs 7 and 19-20, and a possible third site in SCR13 for which evidence remains inconclusive [38,57,58,59]. Polymorphisms or mutations in SCRs 7 and 19-20 may affect interactions of factor H with host cells and basement membranes, and are implicated in the diseases AMD and aHUS (as discussed in section 5).

### 3.3. Factor H Binding to Apoptotic and Necrotic Cells

To maintain tissue homeostasis, old and damaged cells must be removed and replaced by new ones. This is facilitated by apoptosis, a programmed mechanism of cell death, which involves changes such as nuclear and cellular fragmentation, chromatin condensation and cell shrinkage. Changes in the cell membranes facilitate an efficient recognition and safe clearance of apoptotic cells by phagocytes. Complement proteins (e.g., C1q and MBL) and pentraxins (CRP, PTX3) can bind to apoptotic cells and thereby enhance their uptake by phagocytes via specific receptors (*i.e.*, complement and Fcγ receptors) [60]. Moreover, pentraxins can enhance the binding of C1q, which may further increase the deposition of complement-derived opsonins. However, this could potentially lead to the activation of the terminal pathway. Gershov *et al.* showed that complement activation does not proceed to the terminal pathway on apoptotic cells, which is partly due to the binding of factor H [44]. The expression of membrane-bound complement regulators is down-regulated on apoptotic cells, which would increase the susceptibility of these cells to complement-mediated lysis. The loss of membrane-bound regulators on apoptotic cells is in part compensated by the acquisition of the soluble complement regulators factor H and C4b-binding protein, protecting against complement attack which would otherwise lead to the release of potential autoantigens from the cells [45]. The factor H binding site for apoptotic/necrotic cells is located within SCRs 6-20, which is outside the complement regulatory region. Thus surface-bound factor H is able to regulate complement activation. This binding is in part mediated by annexin-II, DNA and histones, which become exposed on the surface of apoptotic cells [46]. In addition, factor H may be recruited by monomeric CRP, and this interaction further facilitates the removal of apoptotic cells in a non-inflammatory way [41]. Notably, native pentameric CRP does not enhance the binding of factor H to apoptotic or necrotic cells [41,45].

### 3.4. Factor H Interactions with Pentraxins

Pentraxins are recognition molecules of the innate immune system [61]. The classical short pentraxins CRP and serum amyloid P component circulate as pentamers in human plasma. The long pentraxins, including PTX3, PTX4 and neuronal pentraxins, display a more complex structure. The functions of pentraxins in innate immune defense and beyond are reviewed elsewhere [61,62]; here, we focus on their interaction with factor H.

*C-reactive protein*. Human CRP is an acute-phase protein, whose synthesis by hepatocytes is up-regulated in response to inflammatory stimuli. Its plasma concentration can increase dramatically from below 1 µg/mL to more than 500 µg/mL following the initiation of an acute phase reaction [63]. The main effector functions of CRP are the activation of the complement system and the stimulation of phagocytosis [64]. CRP activates the classical and lectin complement pathways by binding C1q and ficolins and thus can lead to an enhanced opsonization of target surfaces and cells [65,66]. CRP can be recognized directly by Fcγ receptors on leukocytes, thereby activating phagocytosis [67]. Thus, CRP and complement can collaborate and have synergistic functions, for example in the removal of apoptotic cells and particles [44,60]. However, there are contradictory reports regarding CRP interactions and functions that are in part explained by different conformations of the used CRP [63]. CRP circulates in plasma as a pentamer, whose conformation is stabilized by calcium ions. *In vitro* CRP immobilization on surfaces, such as microtiter plates used for ELISA or chips used for surface plasmon resonance studies, or the use of inappropriate buffer conditions (e.g., low calcium content), may cause denaturation or aggregation of the protein, leading to artefacts or results with no physiological relevance [42,68]. On the other hand, it cannot be excluded that under certain conditions, for instance the lower pH of inflammatory sites, or by binding of the native pentamer to certain ligands and surfaces, conformational changes may occur leading to the exposure of novel epitopes or to the dissociation into the monomeric form, termed mCRP [69,70,71]. These considerations are relevant for assessing the *in vivo* significance of the interaction of factor H with CRP.

A direct binding of factor H to CRP was described [40,72], suggesting regulation of CRP-mediated complement activation on self surfaces. As we and others have shown, factor H mainly interacts with the monomeric or denatured form of CRP [41,68,73,74], although an interaction of factor H with native pentrameric CRP at acute phase concentrations was also demonstrated by analytical ultracentrifugation [42]. Thus, it is unlikely that under normal conditions CRP would interact with factor H to a significant extent. However, under infection/inflammatory conditions (e.g., during the acute phase response or at sites of tissue damage and local inflammation), CRP can bind factor H and locally focus its complement inhibitory activity. The interaction of factor H with mCRP leads to factor H recruitment, which limits complement activation but increases phagocytosis of apoptotic cells and reduces the release of inflammatory cytokines by macrophages [41]. Such regulation is reduced by the common factor H variant 402H, which shows a reduced binding to mCRP on self surfaces [71]. 

*Pentraxin 3*. Aside from the short pentraxin CRP, factor H was also shown to bind to the long pentraxin PTX3 [43]. PTX3 has a pentraxin domain, homologous to that of the short pentraxins CRP and serum amyloid P, and has an additional unique N-terminal domain. In contrast to the short pentraxins that are mainly produced in the liver and thus act systemically in the body, PTX3 is produced locally by various cell types, such as vascular endothelial cells, fibroblasts, monocytes, macrophages, myeloid dendritic cells and neutrophil granulocytes [61,62]. PTX3 expression is increased upon inflammatory stimuli and exposure to pathogens, leading to significantly elevated PTX3 plasma levels (200-800 ng/mL, compared with approximately 2 ng/mL in normal plasma) [62]. PTX3 can activate the classical and lectin complement pathways by binding to C1q, MBL, M-ficolin and L-ficolin [75,76,77,78]. In addition to these complement-activating molecules, PTX3 binds the complement regulators factor H and C4b-binding protein [43,79]. The binding of factor H to PTX3 requires the presence of calcium and is mediated by at least two binding sites in factor H. The primary binding site located within SCRs 19-20 of factor H interacts with the N-terminal domain of PTX3, whereas a secondary binding site on SCR 7 binds to the C-terminal pentraxin domain [43]. PTX3 recruits both factor H and C4b-binding protein to the surface of apoptotic cells, which prevents excessive complement activation and cell lysis [43,79]. We also found that ECM-bound PTX3 increases the recruitment of factor H and C4b-binding protein, resulting in enhanced local complement regulation [79,80]. According to preliminary data, certain factor H mutations in SCR20 and aHUS-associated autoantibodies impair the binding of factor H to PTX3 and this may result in a reduced local complement inhibition [81].

In summary, these *in vitro* studies suggest that factor H binding to pentraxins is important to limit complement activation and inflammation locally. However, further studies are warranted to determine the relevance of factor H-pentraxin interactions *in vivo*.

### 3.5. Factor H Binding to Extracellular Matrix (ECM)

Extracellular matrices are important components of the extracellular space, providing a scaffold for residing cells, mechanically supporting cellular movements and binding various biomolecules derived from body fluids or nearby cells. ECM components may be exposed to complement during pathological processes, such as injury to the endothelium or to the cartilage in the joints. Since the ECM can activate complement, e.g., via the binding of C1q to certain ECM components, a proper regulation is important to prevent inflammation [34]. Because ECMs lack the membrane-bound complement regulators that normally protect host cells, attachment of fluid phase regulators to ECM is considered important. It was shown that the ECM proteins fibromodulin, chondroadherin and osteoadherin can bind both C1q and the regulators factor H and C4b-binding protein, which maintains a balance between complement activation and inhibition [34,47,49]. Exaggerated complement activation in turn may lead to inflammatory disease.

We have recently analyzed complement activation and the roles of factor H and C4b-binding protein on endothelial cell-derived ECM *in vitro*. ECM-bound factor H and C4b-binding protein acted as cofactors for the inactivation of C3b and C4b, respectively. Furthermore, their binding and thus cofactor activity were enhanced by PTX3 [79,80]. The possibility that factor H may play a role in the regulation of local inflammation at the ECM *in vivo* is supported by reports showing factor H binding to the Bruch’s membrane in the retina [82] and an association of factor H with aortic ECM [83].

## 4. Redundant and Non-Redundant Functions among Factor H Family Proteins

The factor H protein family includes factor H, factor H-like protein 1 (CFHL1) and five factor H-related proteins (CFHRs). All these proteins are composed of different numbers of SCR domains, each exhibiting varying degrees of sequence similarity and displaying diverse, but also overlapping, biological functions (reviewed in [8]).

CFHL1 is a 43-kDa protein that derives from an alternative splice product of the *CFH* gene. It shares the seven N-terminal SCRs with factor H and has four additional amino acids. Due to the broad sequence overlapping, CFHL1 possesses the same complement regulatory activities mediated by the N-terminus of factor H. CFHL1 may also play a role in age-related macular degeneration as the SCR7 harbors the Y402H polymorphism. Consequently, the CFHL1 402H variant has impaired ligand-binding capacity, similar to that exhibited by the factor H 402H variant [71,84]. However, an expression pattern differing from factor H and a distinct role in mediating cell adhesion have been reported for CFHL1 [85,86].

The five factor H-related proteins are derived from separate genes (*CFHR1* to *CFHR5*) which are located adjacent to the *CFH* gene. CFHR proteins lack the complement regulatory activities of factor H, but some of them have weak cofactor or decay accelerating activity, or modulate the complement regulatory activity of factor H [8]. These proteins have both redundant and nonredundant ligands and functions with factor H. CFHR1, CFHR3, CFHR4 and CFHR5 were shown to bind to C3b, CFHR1, CFHR3 and CFHR5 to heparin, and CFHR4 and CFHR5 to CRP. In spite of similar cell and ligand binding properties, however, CFHR1 was reported to regulate the terminal complement pathway [87]. In addition, we showed that both factor H and CFHR1 enhance neutrophil adhesion and activation during host cell-pathogen contact [88]. Similar to factor H, CFHR4 binds to late apoptotic and necrotic cells, but in contrast to factor H, CFHR4 binds to the native pentameric form of CRP [68]. Even though a comprehensive knowledge of CFHR functions is lacking, the available data indicate that CFHR proteins may be relevant in regulating local inflammatory processes and could modulate the functions of factor H, e.g. through competition [8].

## 5. Factor H-Associated Diseases

Complement regulatory defects due to factor H mutations or anti-factor H autoantibodies have been described in certain pathological conditions, and *CFH* polymorphisms have also been associated with disease. Here, we briefly review the three diseases where the role of factor H has been best studied. 

### 5.1. Age-Related Macular Degeneration

AMD is a leading cause of visual impairment in elderly, western populations. In recent years, complement gene mutations and polymorphisms have been found to be associated with AMD, pointing to a role of the complement system in the pathogenesis of the disease [89]. Although the underlying pathomechanism is not yet fully known, a role of complement-mediated inflammation in the eye is postulated. Correspondingly, several therapeutic compounds targeting the complement system are currently evaluated in clinical trials [89].

The common factor H polymorphism 402H has been identified as a major genetic risk factor for developing AMD [90,91,92,93]. In addition, a protective *CFH* haplotype associated with the deletion of the *CFHR1* and *CFHR3* genes in AMD has been described [94]. Functional analyses of the factor H 402Y and 402H variants revealed a reduced binding of the AMD-associated 402H variant to mCRP [48,71,84,95,96,97,98]. Since residue 402 in SCR7 is involved in the glycosaminoglycan-binding site of factor H, there are also subtle differences between the variants in their interaction with heparin and glycosaminoglycan-analogs [97,99,100]. In contrast, the 402H variant has a higher affinity for DNA and necrotic cells compared to the 402Y variant [48]. No difference in binding to retinal pigment epithelial cells was found [98], but the disease-associated variant binds less efficiently to both the extracellular matrix protein fibromodulin [48] and the Bruch’s membrane in the retina [82]. In addition, malondialdehyde, a lipid peroxidation product has been described as a novel ligand of factor H on apoptotic/necrotic cells, and shown to bind the 402H variant less strongly, thus adversely affecting the anti-inflammatory role of factor H [50]. Very recently, the rare factor H variant R1210C, previously described in aHUS patients, has been linked to AMD [101]. This variant was shown to affect factor H interactions with C3b and cell surfaces [102]. Altogether, these data suggest a factor H-associated defect in the proper, non-inflammatory handling of cellular waste and in the control of complement activation and inflammation locally at the surfaces of the Bruch’s membrane and damaged retinal pigment epithelial cells. It is still unknown how these factor H defective functions cause or contribute to the late-onset disease AMD in affected individuals, and which other factors (genetic, environmental, life-style) influence the role of factor H. Recent data indicate that common polymorphisms in factor H, C3 and factor B act collaboratively in determining complement activity and the risk to disease [103].

A further functional impairment of the 402H variant is a reduced binding to streptococcal M6 protein [96,98]. Functional studies showed a decreased binding of the 402H factor H variant to *Streptococcus pyogenes*, resulting in increased C3b deposition and phagocytosis [104]. A genetic association study suggested that the 402H variant is protective against streptococcal tonsillitis [105]. These results are highly interesting and indicate that the 402H variant has been established in the human population due to selection pressure by pathogenic microbes.

A study investigating the prevalence of anti-factor H autoantibodies in AMD showed that it is decreased in the patient group compared with age-matched controls. Analysis of the antibody binding sites demonstrated recognition of several parts of factor H, including both the N- and C-terminal domains of the molecule, a pattern different from that seen in atypical hemolytic uremic syndrome (see below) [106]. Therefore, it is unlikely that autoantibodies to factor H have pathological significance in this disease.

### 5.2. Atypical Hemolytic Uremic Syndrome

The rare kidney disease aHUS is characterized by hemolytic anemia, low platelet count and impaired renal function [107]. Its pathomechanism is related to dysregulation of the alternative complement pathway, caused by polymorphisms, mutations and deletions in complement genes, or due to factor H autoantibodies (reviewed in [108]).

Factor H mutations affect approximately 30% of aHUS patients. More than 100 factor H mutations have been described in aHUS patients and can be searched in an online database (http://www.fh-hus.org) [109]. In most cases, these are heterozygous mutations affecting various domains of factor H. However, most of the mutations affect the C-terminal SCRs 19-20. Functional analyses of several of these mutants showed an altered interaction with C3b, heparin and endothelial cells [102,110,111]. Furthermore, gene conversion and gene deletions leading to hybrid factor H proteins with functionally affected C-terminal domains have been reported [112,113,114]. These data show a disturbance in the physiologic interaction of factor H with host endothelial cells. Recent structural studies have provided new insights into how these mutations impair the function of factor H in host-nonhost discrimination [55,56]. Certain mutations in SCR20 were also found to reduce the binding of factor H to CRP [41] and, according to our preliminary data, to PTX3 [81]. For many mutations, however, there is no functional effect known to date. 

Anti-factor H IgG autoantibodies are detected in approximately 10% of aHUS patients [115,116]. This form of aHUS affects mainly children and young patients. As a result of its autoimmune nature, it requires a therapy that addresses the elimination or suppression of the autoantibody producing cells. A further characteristic of this patient group is that 90% of the affected individuals lack the *CFHR1* gene, indicating that this genetic defect predisposes to the development of factor H autoantibodies [116,117,118,119]. These autoantibodies can also occur together with mutations in the *CFH*, *CFI*, *C3* or *MCP* genes [119]. We and others have determined the antibody binding sites in several patients using recombinant factor H fragments and found that the autoantibodies mainly bind to SCRs 19-20 of factor H, although in some cases reactivity with other domains, such as SCRs 8-11, was also observed [116,119,120]. In three patients anti-factor H IgA autoantibodies were found that similarly recognized SCRs 19-20 [121]. Our functional studies indicated that the autoantibodies interfere with the recognition functions of factor H, namely, impairing its interaction with surface-bound C3b and inhibiting the factor H complement regulatory activity on host surfaces [120,121,122]. This reduced protection from complement-mediated damage is likely to be involved in the endothelial injury associated with aHUS. Due to the similar C-terminal SCRs of factor H and CFHR1, most of the studied autoantibodies recognize both host complement regulators. CFHR1 in fact can hijack autoantibodies and rescue host cells when added to anti-factor H autoantibody-positive plasma [121].

Altogether, these genetic and acquired abnormalities affecting factor H allow a normal fluid-phase regulation, but result in an impaired cell binding and cell surface protection from complement attack, which apparently contribute to the endothelial damage and microvascular thrombus formation in aHUS. However, further studies are needed to understand the role and relevance of mutations affecting other domains of factor H in aHUS. A recent study showed that some of the mutations do not lead to any known functional effect on factor H, thus care should be taken when interpreting genetic data and advising patients [123].

### 5.3. Dense Deposit Disease

Dense deposit disease (DDD), also termed membranoproliferative glomerulonephritis type II, is a rare renal disease that progresses to end-stage renal failure in about 50% of patients. It is a disease associated with uncontrolled alternative pathway activation in plasma that generates C3 activation fragments depositing in the glomeruli [124]. In the majority of patients, autoantibodies against the C3 convertase (C3 nephritic factor) can be detected that stabilize the convertase and thus cause enhanced complement activation. In some cases, factor H mutations have been identified in these patients [6,125]. These mutations may lead to factor H deficiency and thus an insufficient plasma complement control [126]. Mutations in cysteine residues that are important for forming the disulfide bonds within the single SCR domains can result in a defective protein folding and a factor H secretion defect [127]. In one report, a C431Y exchange was described that caused aggregation of factor H and likely resulted in a reduced protein half-life [128]. Moreover, a mutation in SCR4 was described, where the mutant factor H protein was inefficient in its cofactor activity, while cell-binding functions remainedunaffected [129]. All these cases led to a defective C3 activation control in plasma, either due to a quantitative factor H deficiency or dysfunctional factor H.

Anti-factor H autoantibodies have also been described in DDD. So far, only one case has been published where the autoantibody was characterized in detail. The isolated factor H ‘mini-autoantibody’ consisted of lambda light-chain dimers that bound to SCR3 of factor H, *i.e.*, within the complement regulatory region of the molecule. Functional assays demonstrated that the autoantibody inhibited the factor H-C3b interaction and caused an increased C3 turnover due to a blockade of the complement inhibitory activity of factor H [130,131]. Due to the lack of systematic screening for such autoantibodies in DDD patients, at present the prevalence and the characteristics of DDD-associated anti-factor H autoantibodies are not known.

## 6. Misuse of Factor H by Pathogens and Tumor Cells

Besides its physiologic interactions with host cells and ligands, the binding of factor H to several pathogenic and non-pathogenic microbes was demonstrated as a process believed to help microorganisms in complement evasion. In other words, these microbes hijack factor H to camouflage themselves as host-like cells and thus are protected from complement attack. Furthermore, certain tumor cells can also exploit factor H to increase their protection from the complement system.

### 6.1. Factor H Binding to Pathogens

Since complement plays an important role in protection against infections, it is not surprising that numerous viruses, bacteria, fungi and parasites have acquired the ability to sequester host complement regulators such as factor H. A detailed discussion of these mechanisms is beyond the scope of this review and there are excellent overviews of this topic throughout the literature (e.g., [35]). As a common theme, it appears that many of the microbial factor H binding proteins interact with the positively charged surfaces on SCRs 7 and 19-20, *i.e.*, the factor H domains relevant for host cell recognition, although for example the factor H binding protein (fHbp) of *Neisseria meningitidis* binds to SCR6 [132]. Interestingly, recent studies indicate that the factor H 402H polymorphism may relate to a better resistance from certain bacterial infections. Haapasalo *et al.* showed that the AMD-associated factor H 402H variant has a lower binding affinity to various streptococci compared to the 402Y variant, resulting in a more efficient opsonization and phagocytosis [104,105]. These data point to a pathogen-driven establishment of this common polymorphism in the human population, with evolutionary advantage against bacterial infection at the expense of late-age adverse effect in developing AMD. This example raises the possibility that other factor H polymorphisms have similarly spread in the human population because of the evolutionary race between humans and their pathogens.

While sequestration of host factor H is described for many pathogens, a biologically relevant role under physiological settings has rarely been demonstrated [105]. Both pathogenic and non-pathogenic microbes can bind factor H, thus it does not necessarily play a decisive role in microbial immune escape. However, microbes may use factor H for other purposes than complement inhibition, such as mediating entry into host cells (discussed below, see section 7).

### 6.2. Factor H and Tumor Cells

Tumor cells have various means to escape from the control of the immune system. Protection from the attack of the complement system is part of this immune evasion repertoire [133]. In addition to modulating the expression of membrane-anchored complement regulatory proteins and degrading complement components, tumor cells may also use factor H for efficient protection [134]. Several tumor cells were reported to express and release increased amounts of factor H, thus reducing complement activity in their microenvironment [135,136,137,138]. The increased factor H expression may even be used as a diagnostic marker for certain cancers [139,140]. Furthermore, in certain cancers the increased expression of the factor H binding proteins: bone sialoprotein, osteopontin and dentin matrix protein-1 may confer enhanced protection from complement [141]. In addition, anti-factor H autoantibodies have been described in non–small cell lung cancer [142]; it is not yet known, however, whether this is due to an increased production of factor H and if and how these autoantibodies differ from those described in aHUS and DDD. These data clearly indicate that tumor cells can exploit factor H to their advantage. Therefore, a targeted inhibition of factor H overexpression by these cells may enhance their sensitivity to complement-mediated lysis.

## 7. Factor H in Cellular Interactions: Beyond Its Role as a Complement Regulator

When bound to the surface of host cells via C3b/C3d and polyanionic molecules, the major task of factor H is to control complement activation. However, factor H can also bind to cell surface receptors and modulate cell activation and cellular functions. To date, studies mainly focused on factor H interactions with soluble (plasma) ligands and their role in complement regulation, but there is a lack of detailed information on factor H interactions with host cell receptors. However, there is support for such non-canonical roles (*i.e.*, beyond its complement regulatory function) of factor H.

Early studies have shown that factor H binds to human B lymphocytes and stimulates a calcium-dependent factor I release from these cells [143], and that factor H also stimulates murine B cells and triggers blastogenesis [144]. Tsokos *et al.* demonstrated that factor H blocks the differentiation, but not the proliferation, of B cells [145]. Attempts to identify the B cell factor H receptor resulted in the description of a putative receptor consisting of three subunits (each of ca. 50 kDa) [146] and in a 140-kDa single polypeptide chain protein [147]. However, the nature of these receptors at the molecular level remains unresolved.

Factor H binds to human neutrophil granulocytes via complement receptor type 3 (CR3; CD11b/CD18, αMβ2 integrin, Mac-1) [148,149]. Factor H was shown to support neutrophil adherence and to enhance the release of reactive oxygen species in primed neutrophils [149]. Recently, we characterized the interaction of factor H with neutrophils in the context of host-pathogen interaction [88]. Factor H, when bound on the human-pathogenic yeast *Candida albicans*, served as a bridging molecule to enhance the adherence and antimicrobial activity of neutrophils. We confirmed CR3 as the major neutrophil factor H receptor, but the data also indicated that CR4 likely binds factor H. Although the latter is expected to play only a minor role in the case of neutrophils, because of low CR4 expression, it might be relevant on other cells, such as macrophages and dendritic cells which express significant amounts of CR4. In addition, we have identified the factor H SCR7 and SCRs 19-20 domains as major binding sites for CR3. The two factor H family proteins CFHL1 and CFHR1 also bound to CR3 and supported neutrophil adhesion [88]. In parallel, other groups have reported that binding of factor H facilitates the entry of pathogens into host cells. The factor H-CR3 interaction could enhance *Streptococcus pneumoniae* adherence and uptake by epithelial cells and neutrophils [150]. Similarly, factor H facilitated the adherence of *Neisseria gonorrhoeae* to human CR3-transfected cells [151]. These data indicate a role for factor H in cellular adhesion by interacting with CR3, even beyond pathogen-host cell interactions. For example, factor H was shown to bind to heparan sulfate proteoglycans in amyloid-β plaques and to colocalize with CR3, suggesting that factor H may facilitate the recognition of amyloid-β plaques by microglia in the Alzheimer’s disease brain [152].

Factor H was shown to stimulate the respiratory burst [153] and to induce the secretion of IL-1β in monocytes [154]. Thrombin-cleaved factor H was described as a monocyte chemotactic factor in a delayed-type hypersensitivity model [155]. A monocyte chemotactic effect of factor H was also demonstrated by Nabil *et al.* [156]. Furthermore, factor H was shown to induce the release of prostaglandin E and thromboxane from guinea pig peritoneal macrophages [157]. In these studies, however, no specific factor H receptor was identified. Since these cells express CR3, it is likely that at least some of the observed effects are mediated via factor H binding to CR3. A recent study and our unpublished data also show that CR3 is a factor H receptor on monocytes [158,159].

Furthermore, the binding of factor H to L-selectin was reported, and immobilized factor H (but not soluble factor H) induced TNFα release from leukocytes [160].

Finally, factor H binds to resting platelets and this binding is increased when platelets become activated [161]. Factor H binds to platelets both through thrombospondin and directly via the platelet integrin αIIbβ3 [162].

Altogether these data attest to potential non-canonical roles of factor H in mediating cellular functions and interactions. These functions and interactions are likely to be important under local inflammatory conditions, such as the regulation of neutrophil adherence and migration, and in the activation of macrophages and dendritic cells. In addition, the interaction of factor H with B cells indicates a potential direct modulatory role in adaptive immunity. However, our present knowledge of factor H cellular functions is very limited. Further studies using highly purified and recombinant factor H are needed either to confirm and extend or disprove these observations. In order to better understand the roles of factor H in health and disease, a comprehensive characterization of factor H-host cell interactions and an assessment of their biological relevance are necessary. It is possible, for example, that known polymorphisms or mutations influence these cellular functions of factor H, providing new insights into the pathomechanisms of factor H-associated disease and revealing novel therapeutic intervention points.

## 8. Conclusions

More than 45 years after its initial discovery, factor H still has many secrets to reveal. The last decade has brought with it a wealth of new information on factor H structure and function, and its pivotal role in host-nonhost discrimination is now well appreciated. We have started to understand how *CFH* variants and autoantibodies are involved in diseases such as AMD, aHUS and DDD. In addition, factor H may play an important role in other common diseases such as asthma [163]. A therapeutic use of factor H in such conditions needs to be evaluated in future studies, which could be facilitated by the recently described recombinant production of biologically active factor H protein [164,165]. In addition to its role as a main regulator of the complement alternative pathway, recent studies show a role for factor H in modulating classical pathway activation by competing with C1q for binding to the same ligands such as phospholipids or *Escherichia coli* [166]. Here again factor H appears as a downregulator of inflammatory responses. Importantly, factor H is not only a complement regulator, but also a direct modulator of cellular functions by binding to receptors. In this role factor H influences cellular adhesion, phagocytosis and antimicrobial activities [88,158]. These aspects of factor H functions warrant further attention as it is likely that they are relevant for innate resistance against infections, the handling of apoptotic cells and debris, modulation of adaptive immunity and cellular interactions with the ECM (potentially including tumor cells). 

**Figure 3:**
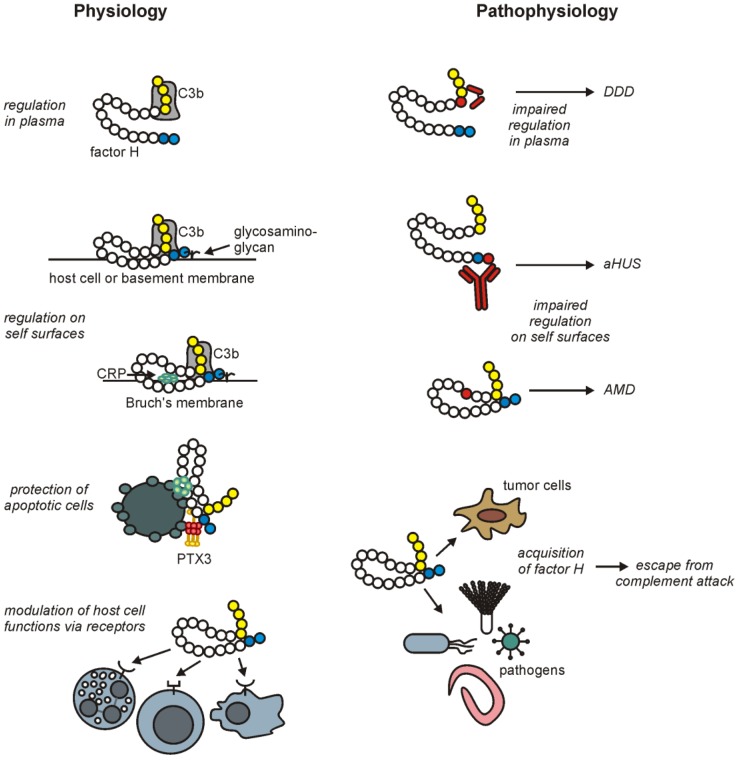
Overview of main factor H functions and their implication in pathological conditions**.** Factor H is a major soluble complement regulator that inhibits activation of the alternative complement pathway in body fluids. In addition, factor H is also able to control complement activation on self-surfaces and thereby protects host cells and tissues from complement attack. Factor H binds to host cells and basement membranes via interactions with glycosaminoglycans and deposited C3b. The binding of factor H to apoptotic cells and extracellular matrices is in part mediated by the pentraxins CRP and PTX3, and is of particular importance as these structures and cells are otherwise not well protected from complement. Furthermore, factor H interacts with host cells via specific receptors and thus modulates cellular functions, including adhesion and phagocytosis. Impaired functions of factor H are associated with several diseases such as DDD, aHUS and AMD. Mutations in the *CFH* gene (examples of affected domains are shown in red) or autoantibodies directed against factor H (“mini-autoantibody” in DDD and anti-factor H IgG in aHUS are shown in red) cause defective complement regulation and ligand recognition leading to improperly controlled inflammation and tissue damage. On the other hand, the regulatory functions of factor H are abused by several pathogens and tumor cells in order to protect themselves from complement attack and thus to evade the host immune response.

Further efforts to identify and characterize factor H ligands, cellular activities and its roles in diseases will bring us closer to a better understanding of the versatile roles played by factor H in both health and disease (Figure 3), with the hope of applying this knowledge for the benefit of all.
